# Temperature stability and enhanced transport properties by surface modifications of silica nanoparticle tracers for geo-reservoir exploration

**DOI:** 10.1038/s41598-024-70132-z

**Published:** 2024-08-19

**Authors:** Laura Spitzmüller, Jonathan Berson, Thomas Schimmel, Thomas Kohl, Fabian Nitschke

**Affiliations:** 1 Geothermal Energy and Reservoir Technology, Institute of Applied Geosciences, Karlsruhe Institute of Technology, 76131 Karlsruhe, Germany; 2 Material Research Center for Energy Systems (MZE), Institute of Nanotechnology, Institute of Applied Physics, Karlsruhe Institute of Technology, 76131 Karlsruhe, Germany

**Keywords:** Tracer, Nanoparticle tracer, Nanoscience, Geoscience, Surface modifications, Nanoscale devices, Other nanotechnology, Nanoscale materials, Nanoparticles, Organic-inorganic nanostructures, Nanoscale materials, Geothermal energy, Geochemistry, Nanoscience and technology, Geochemistry, Hydrogeology

## Abstract

Tracer tests are an important tool for characterizing and monitoring subsurface reservoir properties. However, they are limited both because of the tracer molecules constraining factors such as irreversible adsorption, retention, and degradations, i.e. interaction processes of fluorophore molecule with surrounding media resulting in a large variation in transport properties. Elaborate tests utilizing more than one tracer to distinguish time or location of injection are complex and interpretation is ambiguous because each tracer interacts differently. In this study, we present an approach to increase tracer stability and enhance the transport uniformity of different tracers, thus making tests utilizing multiple tracers simpler and more feasible. We present this concept of tracer multiplicity by encapsulating an anionic, cationic or amphoteric fluorophore inside mesoporous silica nanoparticle carriers coated with a protective titania layer. Upon encapsulation, increased thermal resistance and drastically lowered sorption affinity towards quartz sand was detected in batch and flow-through experiments. An additional advantage of the presented nanoparticle tracers over molecular tracers is their modularity, which is demonstrated by surface modifications and application of additives that greatly reduce sorption and increase recovery rates in the flow experiments. With the here presented concept of tracer multiplicity, we introduce a new approach for colloidal tracer design that has the potential to expand and enhance measurable parameters, measurement accuracy and simplicity of analysis.

## Introduction

The application of molecular fluorescent dyes as tracers is a standard method in hydro- and geosciences to study underground conditions, e.g. fluid pathways, flow direction, flow velocity and interconnections^1–3^. However, their applicability is highly dependent on prevalent fluid properties such as pH-values, fluid temperature and salinity^4^. In fact, the ideal circumstances for fluorescent molecular tracers to behave conservatively are narrowly constrained, that only the sodium salt of fluorescein (uranine) behaves nearly conservative, though only at shallow groundwater aquifer conditions^5,6^. This makes uranine the most used groundwater tracer^7^. Other commonly employed tracers in hydrology are xanthene group dyes, namely eosin Y, sulforhodamine G and rhodamine WT, as well as sodium naphthionate. Cationic xanthene dyes such as rhodamine B and rhodamine 6G are i.a. scarcely used due to their inherent high sorption affinity^8^. For geothermal exploration purposes, prevalent geochemical conditions are harsher than in groundwater, with high salinities promoting sorption^9^, high temperatures degrading the fluorescent molecules^10^ and a wider range of pH-values affecting fluorescence intensity^11^. While photobleaching becomes irrelevant in subsurface, the low temperature stability of most organic dyes limits their applicability^12^. For example, uranine decays above 200 °C in absence of dissolve oxygen^13^, sorption affinity increases under acidic conditions^8^ due to conversion of anionic forms to other prototropic forms of the molecule^14^ and fluorescence is quenched at high salinity^9^. Rose et al.^10^ established therefore the naphthalene sulfonates that can withstand high temperatures^10,12^ as a new class of tracers to overcome the hurdles of geothermal systems. These ultraviolet-tracers (UV-tracers) require sophisticated analysis tools such as HPLC augmenting the analytical cost and effort. Additionally, naphthalene sulfonates tend to isomerize, which has to be taken into consideration during analysis. Moreover, as well as the need to ensure high temperature stability of the tracers to avoid them decaying in the reservoirs, leading to long-term permanence in the produced fluids getting reinjected and hence affect analysis of future tracer tests^12,15^. Other approaches developing new tracers for geothermal include reactive or temperature responsive molecular tracers^16^. Furthermore, within the last years, particle tracers such as quantum dots^17^, nanocrystals^18^, DNA-encapsulated nanoparticles^19,20^ and temperature-responsive fluorescent nanoparticles^21,22^ emerged. This linkage of nanotechnology and geosciences offers new opportunities and possibilities. Nanoparticles tend to stay in main streamlines, having lesser diffusivity and consequently showing faster breakthrough^23^. Recently, the capabilities of DNA-labeled silica nanoparticles and silica encapsulated superparamagnetic DNA tracers were demonstrated in small-scale field applications mainly in groundwater^24–26^. While usage of DNA offers the opportunity of virtually unlimited amount of uniquely identifiable tracers, they are not designed for geothermal applications due to inherent temperature instability of DNA^27^ and solubility of silica^28^. In fact, no silica-based nanoparticle tracer can be successfully applied in aqueous environments unless silica is sufficiently protected against disintegration and dissolution^29^. However, since/because the possibilities/advantages of (silica) nanoparticles tracers outweigh the drawbacks, it is worth developing strategies to overcome these challenges.

In this work we present an approach on a laboratory scale for a nanoparticle carrier that enhances protection of its payload from surrounding conditions and improves uniformity of transport-determining properties between different tracers.

## Concept of nanoparticle tracer and tracer multiplicity

With a limited range of reservoir conditions where tracers are applicable and a narrow variety of tracers that can be used simultaneously^4,8^ augmenting the toolbox of possible fluorescent tracers is needed to make tracer tests more widely applicable and more significant, both for hydrological or geothermal reservoirs. By combining nanotechnology-based approaches with geoscientific research questions, we propose fluorescent dye-encapsulated nanoparticle tracer that fulfills important prerequisites, which are essential for any tracer system that is intended for application in subsurface environments:

- Long-term integrity: Ability to withstand harsh conditions (temperatures, salinities, pH, …) while maintaining functionality
- Identification: The tracers should be clearly distinguishable, easily analyzable e.g. by fluorescence spectroscopy and possibly enable real-time detection
- Favorable transport properties: Tunable retention and/or rock-fluid-tracer interactions

By encapsulating fluorescent dye molecules inside a silica-based nanoparticle carrier, we aim to protect the dye from environmental influences assuring long-term integrity. Consequently, the fluorescence of the dye should not be affected by fluid properties such as pH or salinity. Furthermore, interactions of dye with the surrounding will be limited due to shielding by the carrier; hence, properties of the dye are no longer the decisive factor for transport properties and sorption affinity. This concept of tracer multiplicity enables the possibility of using various uniquely identifiable tracers (by color/fluorescence absorbance/excitation-emission spectra) that have identical transport behavior and sorption properties. An additional advantage of using a particle carrier for transportation of molecular dyes is the modifiable surface of the particle. Whereas transport properties and sorption affinity of molecular tracers are entirely dependent on properties of the molecules themselves such as their charge, which cannot be changed without losing functionality of the molecules, the surface and chemical composition of the nanoparticle carriers can be easily modified to optimize tracer performance. By modifying nanoparticle carrier parameters such as surface charge, surface hydrophilicity and surface roughness, the transport properties can be adapted and controlled without influencing the dye properties.

The herein introduced fluorescent nanoparticle tracers present a feasible approach to tracer multiplicity. In brief, different molecular fluorescent dyes are encapsulated in mesoporous silica nanoparticle carrier capped with an impermeable titania layer^30^. Synthesis and chemical and structural characterization of these nanoparticle tracers is described in a previous study^30^. This study focuses on testing and application of these type of tracers. As investigated in a previous study, pristine silica nanoparticles are not suitable for applications in aqueous environments, as silica nanoparticles are mainly amorphous and consequently soluble in water^29^. However, because silica nanoparticles are a versatile tool, having a highly reproducible synthesis, controllable sizes and porosities, they are still an ideal carrier venue. For applications in aqueous environments long-term (hydro)thermal stability can be achieved by modifying the silica network and/or modifying the surface. Therefore, in the study herein, we base our approach on thermally stable fluorescent nanoparticle tracers (dye-MSN@TiO_2_). The usage of a metal oxide layer overcomes most issues of classical capping agents such as polyethylene glycol (PEG) or other coatings widely used for nanoparticles in biomedical science applications^31–33^ mostly used as reversible pore blockers that can be rendered permeable when triggered with a pH-/temperature-/radiation-stimuli in biological systems but are not robust enough to withstand the more extreme geothermal conditions. In order to maintain particle tracer integrity at high temperatures, calcined mesoporous silica nanoparticle carriers are used to reduce hydrolysis-induced degradation and solubility^29,34^. Addition of a titania layer further increases the (hydro-)thermal stability due to extremely low solubility in water (~ 10^−9^ mol/L)^35^. A detailed description of synthesis and characterization of the dye-MSN@TiO_2_ nanoparticle tracers can be found in Spitzmüller et al.^30^. Three different dyes, the anionic sulforhodamine G (SG, excitation–emission wavelength 532–550 nm), the amphoteric rhodamine B (RhB, excitation–emission wavelength 546–567 nm) and the cationic rhodamine 6G (R6G, excitation–emission wavelength 480–548 nm) are chosen as model dyes for this proof-of-concept investigation as they are comparable but differ in a broad value range in terms of charge and sorption affinity.

This work focuses on examining a laboratory-scale proof-of-concept of the applicability of these nanoparticle tracers. The study consists of three parts: particle characterization, assessment of sorption behavior and particle surface modification. First, we show successful encapsulation and protection of the dye by measuring ζ-potential evolution over a pH-range from pH 1.8 to pH 10.5. By comparing these results to literature data of pristine mesoporous silica nanoparticles and titania nanoparticles, the impact of each individual part (MSN core, dye, titania shell) on the ζ-potential of the dye-MSN@TiO_2_ nanoparticle tracers can be identified. These measurements are used for evaluation of colloidal stability using DLVO force calculations. To evaluate further the protective effect of the encapsulation method, the performance of the particles and the molecular dyes was compared under prolonged exposure to high temperatures (160 °C) in moderately saline fluids (0.1 M NaCl). The fluorescence loss due to high temperatures of the molecular dyes is compared to fluorescence loss of the encapsulated dyes.

The shielding efficiency is further assessed by sorption batch and flow-through column experiments. Hereby, sorption affinity of dyes-MSN@TiO_2_ tracer particles towards quartz sand in 0.01 M NaCl, 0.1 M NaCl and 1 M NaCl solutions and tap-water was compared to the sorption affinity of their respective dyes in the batch experiments. Additionally, flow-through experiments were conducted to examine the effect of particle transport properties and non-static conditions on sorption and retention of the dyes and the encapsulated dyes in 0.01 M NaCl solution.

Finally, we highlight the advantages of tracer particles over molecular tracers, namely the modularity of the particle’s surfaces. By addition of surfactants as well as by chemically modifying the surface, we show the possibilities of the presented concept of nanoparticle tracers. We examined a zwitterionic surfactant (ZI), an anionic surfactant (SDS) and a chelating agent (EDTA) as additives to the fluid matrix and tested their ability to reduce sorption affinity of the nanoparticles towards the quartz-sand surfaces. The anionic surfactant SDS serves to stabilize the colloidal suspension and keeps the particles separate^36^ while EDTA is commonly used to inhibit scaling formation in geothermal brines and are therefore selected as suitable additives^37^. Contrarily to solely adding additives, we also modified the nanoparticle tracers directly by bonding silanes to hydrophobize the surface of the nanoparticles. Having a hydrophobic tracer could be beneficial for example in applications where oil or oil residues might be present and could be of particular interest for oil and gas industry^38^. All these kind of modification strategies are a unique option for colloids and particles, hence expand the possible application range and scope for tracer tests.

## Experimental

**Figure 1:**
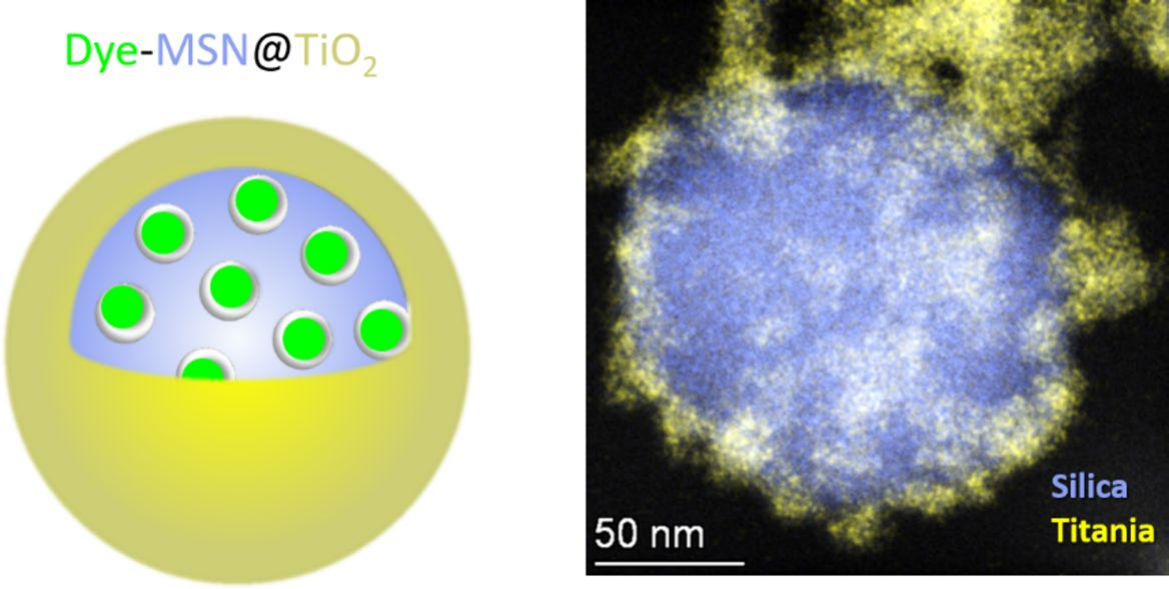
Sketch of the dye-MSN@TiO_2_ nanotracer and elemental mapping image depicting silica and titania. The dye is located within the pores of the mesoporous silica nanoparticle. A titania shell is added as a stable pore blocker and protects the mesoporous silica carrier.

The nanoparticle tracers used in this study are 151 ± 38 nm with a of 142 ± 24 nm mesoporous silica core and a 7.1 ± 4.3 nm thick titania shell determined by SEM and TEM imaging^30^. The dye (R6G, SG or RhB) is located within the 2–3 nm-sized pores in the mesoporous silica. A sketch of the dye-MSN@TiO_2_-nanoparticles and an elemental mapping image depicting silica and titania is given in Fig. 1. In the Supplementary information, the synthesis procedure is briefly described. A more comprehensive study of the whole synthesis process and characterization can be found in Spitzmüller et al.^30^ with detailed structural analyses. Figure S1 (Supplementary file) shows FT-IR-ATR spectra with titania-related, silica-titania-related and dye-related vibration bands and representative SEM images of the particles.

### Analytical devices

#### SEM

A Zeiss Leo 1530 was used for SEM imaging with aperture 30 µm, EHT-voltage of 1–3 kV, and usually working distances of 1.5–3 mm. Samples were prepared following the latter procedure. First, a *p*-doted silicon wafer was placed on top of an adhesive carbon-tap. Then, nanoparticle suspension was diluted with ethanol and 5 µL were dropped to the silicon wafer and dried under nitrogen atmosphere. Because coating of the samples with carbon or gold was avoided, measurements were performed at low EHT voltages in order to not charge the samples. Images were taken in InLens or SE2 mode.

#### ζ potentiometry

To measure ζ-potential of the nanoparticles a Malvern Zetasizer NanoZS and folded capillary cell were used. ζ-potential analyses were performed in 10 mM KCl solutions at 25 °C at pH 1.8, 4.4, 6.6, 8.5 and 9.5. pH was adjusted prior to measurement using HCl or NaOH. pH was measured using pH-meter VWR pH20. Electrophoretic mobility was measured and converted according to Smoluchowski approximation to ζ-potential. Measurement sets were repeated threefold with minimum 10 individual measurements within sets. ζ-potential represents the charge on the shear plane of the particles implying its strong dependence on salinity and pH of the solution. From literature experimental data^39^ and DLVO theory^40,41^ a general decrease of the absolute ζ-potential can be expected with increasing salinity and especially in presence of divalent ions. There are indications of neutralization and even overcharging of the surface^42^. One important information when interpreting ζ-potential data is the location of the isoelectric point (iep). At this pH-value resulting ζ-potential is zero, meaning the surface charge of the particle and the charge of the ions in the Stern and diffuse double layer are equaling their charge. Below that point, ζ-potential is typically positive, whereas above that point it is usually negative. Furthermore, the absolute value of ζ-potential indicates agglomeration tendency or colloidal stability, with rule of thumb of above absolute values of 30 mV suspensions considered colloidal stable^43,44^.

#### FT-IR-ATR

FT-IR ATR analysis was performed using a Nicolet iS50 with wavenumbers from 4000 to 400 cm^−1^. Measurements were repeated twenty times. Samples were measured in dry conditions.

#### Fluorescence

Fluorescence was measured with a Cary Eclipse fluorescence spectrometer using 10 mm × 10 mm PMMA cuvettes for static conditions and quartz glass flow-through cuvette for monitoring breakthrough curves in flow-through experiments. Rhodamine 6G emission spectra were measured with excitation wavelength 480 nm, rhodamine B and sulforhodamine G were measured as synchroscans with Stoke shifts of 20 nm and 18 nm, respectively.

### Sorption experiments

#### Fluid preparation

Batch sorption experiments were performed using stock solutions of NaCl in Millipore water (≥ 18 MΩ) with concentrations of 0.01 M (pH 6.9), 0.1 M (pH 6.7) and 1 M NaCl (pH 5.9). Tap water (pH 6.6) with ionic strength (IS) comparable to 0.01 M NaCl solution was used as retrieved (Table 1). No buffers were used to fix the pH as they would affect the overall ionic strength of the fluids. Instead, the fluids were equilibrated for 24 h with the quartz-sand under ambient atmospheric conditions before pH was measured. Flow-through experiments were performed using 0.01 M NaCl fluid. This low salinity was chosen to be able to perform DLVO-calculations based on measured ζ-potentials that are standardly measured in 0.01 M NaCl or KCl solution. For temperature experiments, 0.1 M NaCl fluids were used.

**Table 1:** Composition of fluids used for sorption and temperature experiments. All concentrations are displayed in mg/L, ionic strength (IS) in mmol/L. pH was measured after 24 h equilibration time of fluid with quartz sand at ambient conditions.

	Tap water^a^	0.01 M NaCl	0.1 M NaCl	1 M NaCl
Na^+^	12.7	230	2300	23,000
K^+^	1.9	< 0.004^b^	< 0.04^b^	< 0.4^b^
Ca^2+^	115			
Mg^2+^	11.1			
SiO_2_	5.7			
Cl^−^	25.8	355	3550	35,500
HCO_3_^−^	333	^c^	^c^	^c^
SO_4_^2−^	49.2			
TDS^d^	558	585	5850	58,500
IS	11	10	100	1000
pH^e^	6.6	6.9	6.7	5.9

^a^Composition according to Stadtwerke Karlsruhe 2022.

^b^< 15 mg K_4_(Fe(CN)_6_)) per kg NaCl as stated by VWR GPR Rectapur.

^c^Due to equilibration with atmospheric CO_2_ HCO_3_^−^ concentration always above 0.

^d^As calculated.

^e^As measured after 24 h equilibration with quartz sand in glass vials.

#### Sorption experiments

Sorption experiments were performed in glass vials. Washed and dried quartz sand (grain diameter 1–1.6 mm, CAS number 14808-60-7) was mixed with the fluid (tap water (TW), 0.01 M NaCl, 0.1 M NaCl, 1 M NaCl, Table 1) in a w/v-ratio of 1:2.5. The solutions are rotated 24 h using a laboratory shaker (IKA rocker 3D) at a speed of 60 rpm to equilibrate the fluid with the quartz sand. After equilibration, molecular tracers are added to reach concentrations of 10^−4^–10^−3^ mg/mL (0.1–1 ppm), particle tracers are added to yield concentrations of 10^−2^–5 × 10^−2^ mg/mL (10–50 ppm). In total, 72 individual experiments were performed: three different molecular tracers and three different particle tracers, each in four different fluid types. For each experiment, an individual min. 4-point fluorescence calibration with the corresponding fluid was measured to correlate fluorescence intensity and concentration. The vials were shaken at 60 rpm while protected from photodegradation by wrapping with aluminum foil. Samples were measured after 1.5 h, 4 h, 24 h, 48 h and 168 h to monitor fluorescence of the fluid. The concentration at each time point (and consequently the amount of tracer lost to sorption) could be calculated using the corresponding calibration curve. To possibly identify and consider effects such as aggregation of nanoparticles and settling of aggregates as removal mechanism (i.e. resulting in lower concentration in the fluid although not induced by sorption) in the evaluation, the calibration points were re-measured at each time point when samples were measured. All experimental sets (dyes with each of the fluids and particles with each of the fluids) were triplicated and individually calibrated to calculate sorption percentage. Three different dyes were used: the anionic sulforhodamine/amidorhodamine G (SG), the amphoteric rhodamine B (RhB) and the cationic rhodamine 6G (R6G). Testing sorption affinity in batch experiments might overestimate sorption affinity due to high residence/contact time of the tracers and particles with the mineral surfaces and due to reversible adsorption^45^. However, testing the properties under least favorable conditions mimics the “worst-case-scenario” and benchmarks the highest sorption affinity.

#### Calculation of K_d_-values

K_d_-values (L/kg) are calculated after 48 h according to Bork et al.^46^

$${K}_{d}= \frac{\left({c}_{0}-{c}_{aq}\right)*{V}_{0}}{{m}_{sample}*{c}_{aq}}$$

with c_0_ (µg/L) initial concentration, c_aq_ (µg/L) concentration in fluid after 48 h, V_0_ (L) initial fluid volume, m_sample_ (kg) sample weight. K_d_ values express sorption affinity with higher values indicating faster and stronger sorption than lower values.

#### Flow-through experiments

**Figure 2:**
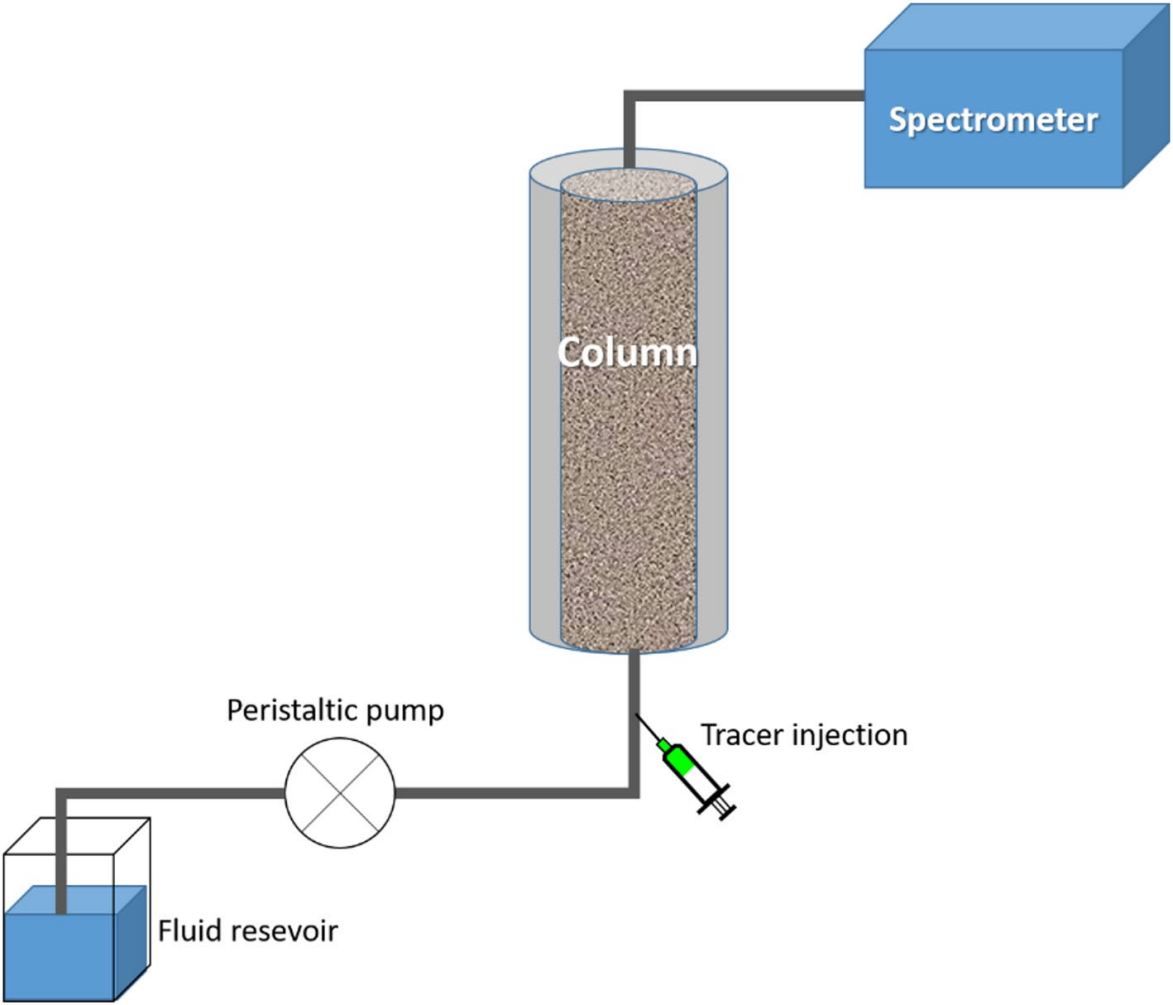
Sketch of the flow-through setup (not true to scale). The fluid used is 0.01 M NaCl solution which is flown through the system with 0.27–0.28 mL/s. A peristaltic pump is used to maintain the flow rate. The tracers are injected through a septum upstream of the column. The column is packed with quartz sand. Produced fluids are measured in a flow-through quartz cuvette in the spectrometer and are wasted afterwards (i.e. not reinjected).

To test the transport ability in flow-through setup, particles and dyes were circulated through a 25 cm PVC column (inner diameter 32 mm) packed tightly with washed and dried quartz sand (approx. 320 g, grain size 1–1.6 mm). To avoid preferential flow paths along the column wall, they were coated with a continuous layer of quartz sand. The inlet and outlet were covered with sinter glass plates (pore size 160–250 µm, VitraPOR Por. 0 ISO 4793-80, ROBU Glasfiltergeräte GmbH Germany). The liquid exiting the column was directed into a quartz flow-through cuvette inside the fluorescence spectrometer, where measurements were performed with a frequency of 1 Hz. The produced fluids were not reinjected into the system. The column was connected to the measurement setup using Festo and silicon pipes of 10 mm and 3 mm diameter, respectively with constant flow rate of 0.27–0.28 mL/s maintained by a peristaltic pump (DüLabo PLP380). Flow direction was from bottom to top in order to avoid air bubbles inside the column. Particles and dyes were injected as one-pulse injection into the pipe upstream of the column inlet with a syringe through a septum. A sketch of the setup is shown in Fig. 2. Concentration was calculated from the fluorescence intensity through intensity-concentration calibration curves. Dye and particles were mixed with 0.01 M NaCl solution to make 10^−3^ mg/mL and 10^−1^ mg/mL solutions, respectively. Flow velocity was kept constant and monitored for each measurement separately. To ensure comparability, the measured time was converted to volume and signals were normalized according to peak intensity. Breakthrough curves were analyzed according the c_peak_-method^47,48^ (Supplementary Information Section 2.1).

### Modification of surface properties

Surface modifications can be performed either by chemically bonding, coupling or grafting molecules like silanes or polymers or by weaker electrostatic interaction with surfactant materials such as sodium dodecyl sulfate (SDS) or cetrimonium bromide (CTAB)^49–51^.

#### Sorption experiments with additives

The impact of different additives on sorption affinity of the particles was tested using the anionic surfactant sodium dodecyl sulfate (SDS), the zwitterionic surfactant SB3-14 (sulfobetaine 3-14, *N*-tetradecyl-*N*,*N*-dimethyl-3-ammonio-1-propanesulfonate, herein abbreviated as “ZI”), and the chelating agent ethylenediaminetetraacetic acid (EDTA). The effective concentration for each surfactant varies, with the concentration used in the herein described experiments being 0.1 mg/mL SDS, 0.1 mg/mL ZI and 5 × 10^−3^ mg/mL EDTA. Sorption tests and calibration curves were performed as described in the previous section. For ζ-potential analyses, samples underwent two washing-centrifugation cycles with Millipore water to remove free additive molecules from the solution, i.e. ζ-potential differences indicate attachment of additives to nanoparticle surfaces.

#### Phase-separation experiments

The surface of dye-MSN@TiO_2_ nanoparticle tracers is hydrophilic, i.e. the particles are easily dispersible in water. By chemically modifying the surface via silanization, the particles were altered hydrophobic, i.e. dispersible in apolar solvent. To demonstrate the effect of surface modifications, phase-separation experiments are conducted in a separation funnel with water being the polar and hexane being the apolar phase. Fluorescence was measured from each of the phases to determine the presence or lack thereof the nanoparticles. Furthermore, the silanization of the surface is proven by FT-IR ATR measurements.

### Thermal degradation

Temperature stability of the dyes and dye-MSN@TiO_2_ was examined at 160 °C for 120 h in 0.1 M NaCl solution with air as head space. Pressure can be approximated to be 6–8 bar. Experiments were duplicated and individually calibrated. Calibration was repeated after 120 h in order to identify aggregation and clumping effects (particles) or degradation in aqueous medium (dye). 20 mL of 10^−4^ mg/mL dye solution and 20 mL of 10^−2^ mg/mL particle-dispersion were placed in teflon liner inside stainless steel autoclaves and heated in an oven at 160 °C under static conditions. After 120 h, the samples were cooled to room temperature before fluorescence was measured. It should be noted that in geothermal environments, no dissolved oxygen is present.

## Results and discussion

### Nanoparticle tracer characterization

#### ζ-potential and DLVO theory

**Figure 3:**
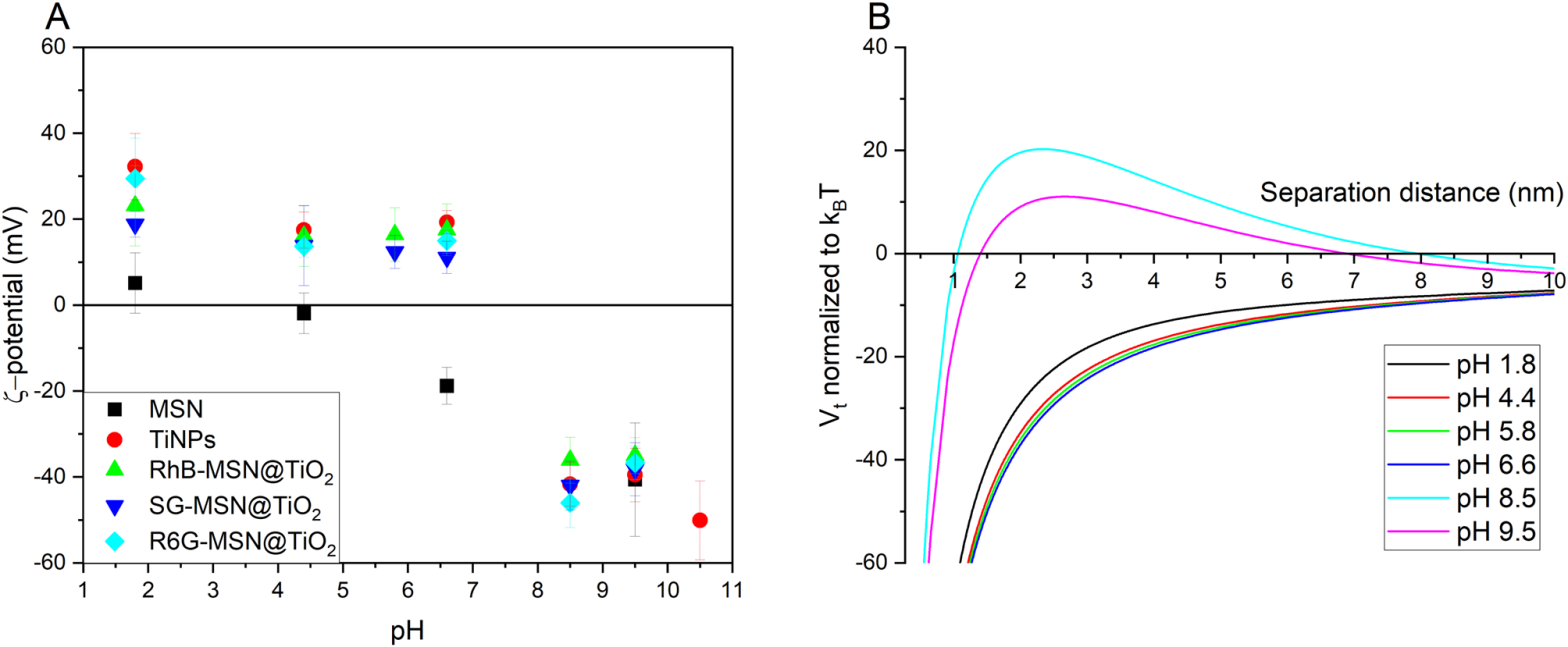
(**A**) ζ-potential over pH in 10 mM KCl solution of pristine mesoporous silica nanoparticles (MSN), titania nanoparticles (TiNPs), and dyes encapsulated in mesoporous silica nanoparticles coated with a titania layer (dye-MSN@TiO_2_). The nanoparticle tracers exhibit zeta potentials in the range of titania nanoparticles, indicating successful surface coating. RhB rhodamine B (amphoteric), *SG* sulforhodamine/amidorhodamine G (anionic), *R6G* rhodamine 6G (cationic). (**B**) Particle–particle interaction energy according to DLVO theory at different pH for SG-MSN@TiO_2_.

Figure 3 depicts ζ-potential evolution over pH-value for pristine MSN carrier, titania nanoparticles, and dye-MSN@TiO_2_ tracer nanoparticles and compares impact of ζ-potential on surface interactions described by DLVO theory. The isoelectric point (iep) of MSN lays in the range between pH 1.8 and 4.4, in agreement with literature data^39^. The negative charge at neutral pH-values correlates to low sorption affinity of silica nanoparticles towards minerals with negatively-charged surfaces such as quartz. Contrarily, pure titania nanoparticles show their isoelectric point between pH 6.6 and 8.5, corresponding to iep 6.8 in literature^43,52,53^. Below pH 6.6 the ζ-potential is positive, possibly indicating stronger sorption tendency on negatively charged mineral grains. The ζ-potential of dye-MSN@TiO_2_ nanoparticle tracers show comparable behavior to pure titania nanoparticles: their iep is located between pH 6.6 and 8.5 with slightly positive charges in neutral pH-range and highly negative ones in alkaline environment. This finding implies two indications: First, the synthesis did yield a continuous titania coating as intended, otherwise the silica core would have influenced the resulting ζ-potential. Second, the procedure effectively shields the dye from surrounding; the ζ-potentials are similar regardless of the type of incorporated dye (anionic, cationic or amphoteric). In conclusion, the coating layer affects the ζ-potential more significantly than the mesoporous silica core and the encapsulated dye.

Calculation of DLVO interaction energy can be used to estimate the colloidal stability of the suspensions, i.e. whether the particles are prone to aggregation, flocculation and clumping. Figure 3B shows the sum curve of attractive van-der-Waals forces and repulsive electric double layer (EDL) forces calculated for SG-MSN@TiO_2_ nanoparticles (Supplementary Information Section 2). Hereby, positive values represent repulsive forces while negative values are attractive. In close proximity, the attractive van-der-Waals forces are predominant leading to a primary minimum, i.e. if the separation distance of the particles is below that value then the particles aggregate irreversibly^54^. EDL forces highly depend on the ionic strength of the solutions and the surface charge of the particles. From the force curves in Fig. 3B it can be seen that alkaline pH-values stabilize colloidal suspensions due to higher absolute ζ-potential and hence higher repulsive force between the nanoparticles. Contrary, near neutral ζ-potentials are not sufficient to overcome the attractive van-der-Waals forces resulting in coagulation and flocculation. The energy barrier can be used to determine the rate of coagulation in dependence on the particle concentrations^55^. For example, in a dispersion at pH 8.5 with 1 mg/mL particles, the system is a stable colloidal dispersion for approx. 6500 years (20k_B_T, Supplementary Information Section 2.2.3).

ζ-potentials were measured in 0.01 M KCl solution at 25 °C. However, in geothermal environments, ionic strength usually exceeds 0.01 M and the temperature is higher. Studies with simulated and measured data of ζ-potential evolution of nanoparticles and reservoir minerals with increasing salinity^39,56–58^ generally show the following trend: increasing ionic strength reduces (i.e. neutralizes) the ζ-potential of both nanoparticles and reservoir minerals, thus decreasing the Debye length and absolute values of EDL forces (either attractive or repulsive). Liu et al.^56^ found 1 M NaCl solution to be ζ-potential neutralizing and high Ca^2+^-concentrations even being able to reverse the ζ-potential of silica nanoparticles, thereby strongly affecting both the dispersion stability and the sorption affinity. Estimation of the Debye length yields approximately 3 nm in 0.01 M NaCl, 0.96 nm in 0.1 M NaCl and 0.3 nm in 1 M NaCl (Supplementary Information Section 2).

#### Temperature stability: evaluation of protection and isolation of payload molecules by the encapsulating layer

**Figure 4:**
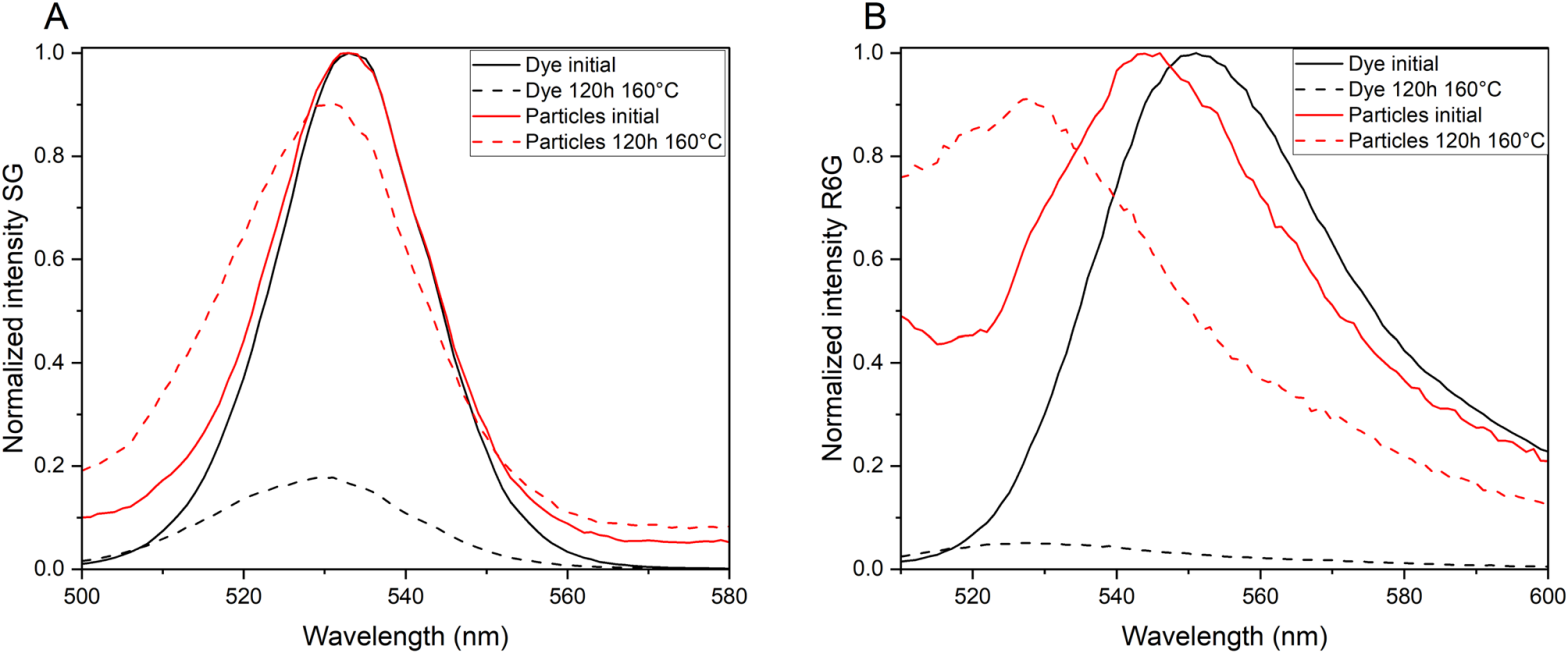
Normalized fluorescence emission spectra of SG and encapsulated dye SG-MSN@TiO_2_ (**A**) and R6G and encapsulated dye R6G-MSN@TiO_2_ (**B**). Spectra shows initial fluorescence intensities and fluorescence after 120 h at 160 °C in 0.1 M NaCl solution. Intensity loss of SG-dye fluorescence 82.3% while for SG-MSN@TiO_2_ fluorescence loss of 8.4% (**A**). Intensity loss of R6G-dye 94.9% and R6G-MSN@TiO_2_ 8.7%, respectively (**B**).

The crucial point for tracer application in high-temperature environments is the fate/degradation of the organic fluorescent dye. Figure 4 shows fluorescence emission spectra of dye and dye-MSN@TiO_2_ before and after heating for 120 h at 160 °C in 0.1 M NaCl solution. Fluorescence intensity decreased by 82.3% and 94.9% for SG-dye and R6G-dye, respectively. Under identical conditions, fluorescence loss of SG-MSN@TiO_2_ is 8.4% and for R6G-MSN@TiO_2_ 8.7%. Hence, protection of the encapsulated molecules is achieved and fulfills one/two of the main prerequisites presented in the introduction for tracer: the long-term integrity under harsher conditions and the identification. Possible explanations for the protective effect could be reduction of degrees of freedom of the encapsulated dye, damping of the thermal energy by the carrier matrix or protection from oxygen. It is known that presence of oxygen accelerates the thermal decomposition of dyes^13^. It can therefore be assumed, that the nanoparticle carrier acts as a barrier and protects successfully the encapsulated dye. Further, it is an indication, that the particles stays intact, otherwise the fluorescence loss would be as observed for the free dyes. SEM images of the nanoparticles after heating can be found in Supplementary Fig. S2.

The peak shift to shorter wavelength in both the spectra of the dye and the dye-MSN@TiO_2_ after heating could be a sign of reduced conjugation of the chemical bonds of the fluorophore or could be due to interactions with the particle matrix^59–61^. The peak shifts of 3–4 nm for SG and SG-MSN@TiO_2,_ 22 nm for R6G-dye and 16 nm for R6G-MSN@TiO_2_ in the experiments were permanent, i.e. even after days at room temperature the peak shift was still present for heated dyes and dyes-MSN@TiO_2_. Implications of these systematic peak shifts could affect interpretation of breakthrough curves and calculation of tracer recovery in geothermal tracer tests since fluorescence measurements are usually performed at fixed wavelengths of fixed Stoke shifts possibly underestimating the peak intensity. Therefore, the excitation and emission wavelengths of tracer dyes should be reassessed at conditions that simulate the thermal exposure endured during geothermal tracer tests, as the resulting shifts may considerably affect the test results.

### Assessment of sorption affinity and transport behavior

The aim of nanoparticle carriers is to protect the encapsulated dye from the surrounding, i.e. dye properties ideally should be independent of the sorption, retention and transport behavior of the nanoparticles. To examine the effect of encapsulation on the interaction of the tracer with the environment, sorption tests were performed comparing sorption affinity towards quartz sand of the dye and dye-MSN@TiO_2_.

#### Anionic dye: sulforhodamine G

**Figure 5:**
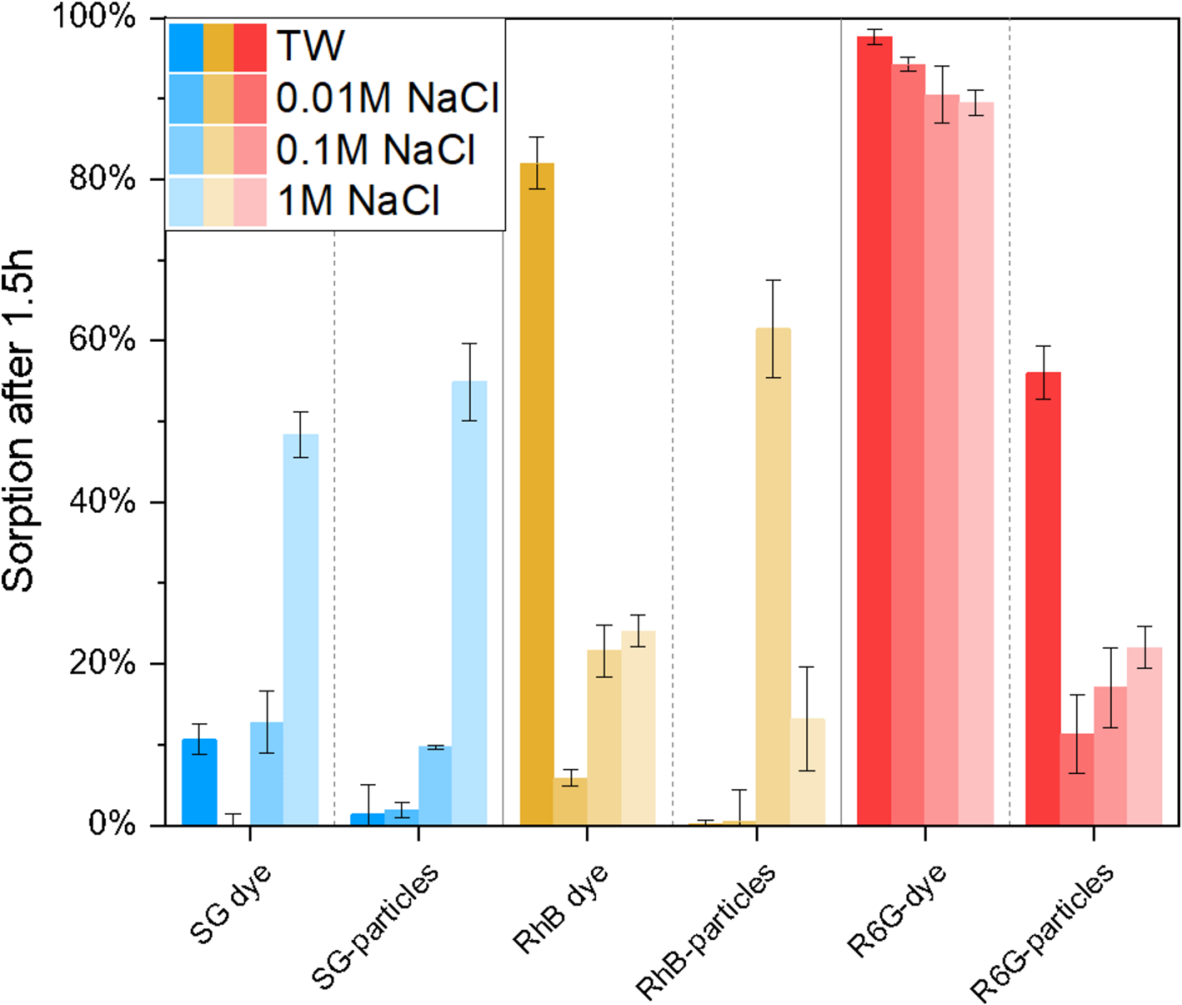
Comparison of sorption percentage in different salinities after 1.5 h reaction time. Experiments were triplicated and individually calibrated. Bluish colors refer to SG Sulforhodamine G (anionic dye), yellowish colors RhB = Rhodamine B (amphoteric dye), and reddish colors to R6G = Rhodamine 6G (cationic dye).

Whereas low sorption affinity of anionic SG-dye towards negatively charged quartz grains can be expected^62,63^, high salinities are known to increase sorption affinity of molecular dyes as their solubility is decreased^9^. As expected, experiments reveal strong sorption dependency on fluid’s salinity, with highest sorption of 48% after 1.5 h in 1 M NaCl solution (Fig. 5). A dependency on ion valence can be assumed due to slightly increased sorption in tap water (calcium-carbonate system, Table 1) with K_d_ 0.19 L/kg compared to K_d_ 0.11 L/kg in 0.01 M NaCl solution (Table 2). Encapsulation of the anionic dye in nanoparticle carriers does not significantly reduce sorption affinity. In NaCl solutions SG-MSN@TiO_2_ behave like SG-dye, presenting strong dependency on fluid’s salinity. In 1 M NaCl sorption affinity is stronger for particles compared to dye molecules (55% compared to 48%). However, in complex fluid (tap water) this behavior is reversed (1% for particles compared to 11% for dye). One explanation for increasing sorption affinity of nanoparticles with increasing salinity can be found in the DLVO-theory. High salinities decrease the electric double layer (EDL) thickness and net charge, hence lowering repulsion forces between particles. Consequently, the particle dispersion tends to destabilize and forms aggregates^64^. However, high salinities also affect the surface charge of quartz, possibly resulting in lowered attractive forces.

**Table 2:** Calculated K_D_-values in L/kg after 48 h interaction time of dye and dye-MSN@TiO_2_ in different salinities.

	Tap water	0.01 M NaCl	0.1 M NaCl	1 M NaCl
SG dye	0.19 ± 0.10	0.11 ± 0.13	0.48 ± 0.11	4.99 ± 0.13
SG-MSN@TiO_2_	0.21 ± 0.06	0.24 ± 0.10	0.65 ± 0.10	10.61 ± 1.89
RhB dye	18.21 ± 11.20	0.24 ± 0.03	0.99 ± 0.05	0.98 ± 0.15
RhB-MSN@TiO_2_	0.42 ± 0.42	0.83 ± 0.32	16.28 ± 8.29	2.38 ± 0.43
R6G dye	227.41 ± 58.54	95.12 ± 32.00	347.73 ± 314.07	349.96 ± 219.50
R6G-MSN@TiO_2_	6.75 ± 2.08	0.58 ± 0.19	1.73 ± 1.29	1.19 ± 0.16

#### Cationic dye: rhodamine 6G

Cationic dyes are unfavorable tracers due to their strong sorption affinity toward negatively charged surfaces such as quartz. As expected, R6G-dye shows high sorption affinity, mostly independent of fluid properties with 90–100% sorption (Fig. 5) and K_d_ values of 95–350 L/kg (Table 2). In contrast, when encapsulated in the nanoparticle carrier, sorption is significantly lowered in NaCl solutions and is only slightly dependent of fluid ionic strength with 11% sorption in 0.01 M NaCl solution and 22% in 1 M NaCl solution (Fig. 5). Highest sorption affinity with 56% is observed in tap water (calcium-carbonate system, Table 1) which could hint a dependency on ionic valence. However, the overall enhanced sorption properties for R6G-MSN@TiO_2_ under all tested conditions can be unequivocally ascribed to encapsulation and effective protection/shielding of the cationic dye.

#### Amphoteric dye: rhodamine B

RhB-dye shows slight dependency on fluid salinity (6% in 0.01 M NaCl to 24% in 1 M NaCl) but stronger dependency on ion valence, i.e. highest sorption affinity in tap water with 82% sorption (calcium-carbonate system) and K_d_ 18.2 L/kg. In comparison, for encapsulated RhB in nanocarriers K_d_ value is reduced significantly to 0.42 L/kg under the same conditions. However, RhB-MSN@TiO_2_ show unexpected strong sorption affinity in 0.1 M NaCl (61%), which could be neither attributed to increasing ionic strength, as sorption affinity in 1 M NaCl is significantly lower (13%), nor to pH-value which was similar to pH of tap water. Consequently, this setup was chosen to assess the effect of additives on sorption affinity.

**Figure 6:**
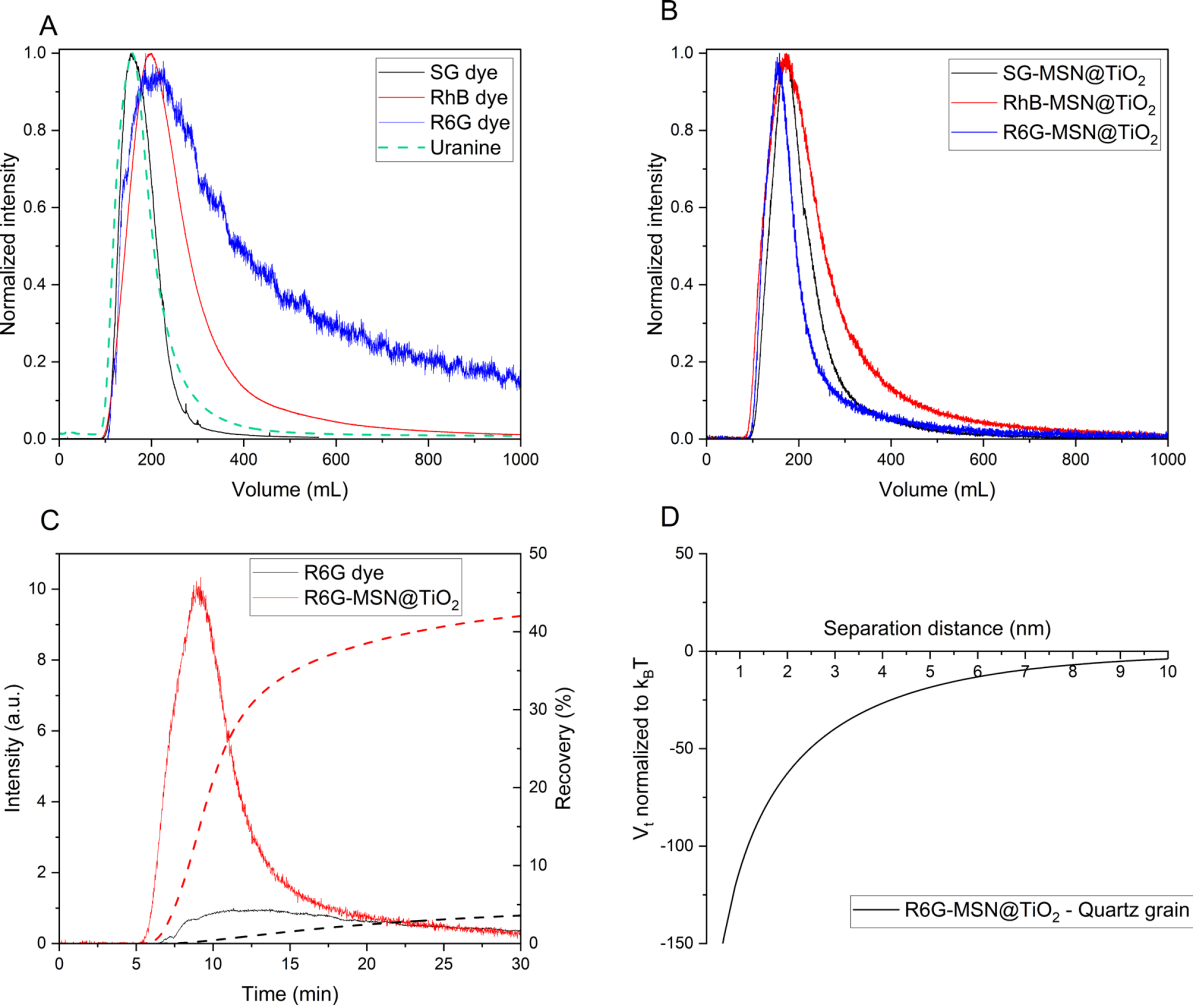
Breakthrough curves of dyes and particles through a 25 cm long column filled with quartz sand at room temperature in 0.01 M NaCl solution and DLVO interaction calculations. A and B compare breakthrough curves of dyes (with uranine being the “ideal” reference tracer) and dye-MSN@TiO_2_ particles, respectively. Intensity is normalized to peak intensity and measured time is calculated to volume by multiplication with flow rate to balance slight differences in flow rates between the experiments. C shows breakthrough curves of R6G dye and R6G-MSN@TiO_2_. Dashed lines represent recovery rate over time. D shows total DLVO interaction energy between R6G-MSN@TiO_2_ and quartz grains in 0.01 M NaCl solution.

Additional to batch sorption experiments, laboratory scale flow-through experiments were performed to test the effect of previously assessed sorption behavior on transport properties. Figure 6 compares breakthrough curves of molecular dye tracers and dye@MSN-TiO_2_ through a 25 cm column filled with quartz sand. As expected from batch experiments, the cationic dye R6G-dye shows sorption and retention (indicated by long tailing) while the breakthrough curve of the anionic SG-dye is nearly ideal (Fig. 6A, uranine as comparison for ideal behavior). For the dyes@MSN-TiO_2_ differences in breakthrough curves are less pronounced, indicating a more uniform transport behavior as compared to their respective dyes (Fig. 6B). Furthermore, direct comparison of R6G-dye to R6G-MSN@TiO_2_ highlights the advantages of particle tracers with faster arrival of particles and shorter peak time values (Fig. 6C). This behavior can be attributed to flow properties of colloids where particles tend to stay in the main streamlines and hence experiencing reduced dispersion^65^. Furthermore, the difference becomes apparent when comparing the recovery: while only 3.5% of the initial amount of R6G dye is retrieved, particle recovery is 42%. Thus, upon encapsulation, recovery is increased by a factor of 12. Contrarily, from DVLO calculations, one can expect highly attractive conditions between dye-MSN@TiO_2_ and quartz grain collectors, which should result in adsorption of the particles and high retention rates (Fig. 6D). However, DLVO calculations omit hydrodynamic forces related to flow-through conditions such as drag and torque forces^66,67^ and surface roughness of the collector grains^68^. These factors, according to the relatively higher particle recovery rate, seem to have a more significant contribution in determining particle transportability. Additionally, another explanation for the higher recovery could be related to the size difference of particles, quartz grains and pore throats. With a size ratio of particles-to-grain of 0.01% below the threshold of 0.5% retention of the particles due to straining is not expected^69^. However, it should be noted that other physicochemical filtering processes could still be relevant^70^. Calculations of dispersivity, dispersion coefficient, and mean velocity show less dispersivity, less dispersion and higher mean velocity of particles compared to dyes (see also Supplementary information Table S2). Dispersivity ranges for particles between 4.55 × 10^−3^ to 1.32 × 10^−2^ m, for dyes between 6.34 × 10^−3^ to 3.25 × 10^−2^ m. Dispersion coefficient ranges for particles between 1.64 × 10^−2^ and 4.28 × 10^−2^ cm^2^/s and for dyes between 2.93 × 10^−2^ to 8.76 × 10^−2^ cm^2^/s. Mean velocity ranges for particles between 3.24 × 10^−4^ to 3.6 × 10^−4^ m/s and for dyes between 2.7 × 10^−4^ to 3.55 × 10^−4^ m/s.

### Modification of surface properties

The sorption tests presented above were performed with the dye-MSN@TiO_2_ tracer without any additives or surfactants. However, unlike molecular dyes nanoparticles offer the unique opportunity to modify their behavior via surface treatment. This can be achieved either by loose electrostatic attraction or by self-assembly of a covalently-bound monolayer to the nanoparticle surfaces.

#### Additives

To test the ability of tuning sorption affinity by simply modifying nanoparticle surface and net surface charge, the system 0.1 M NaCl solution + RhB-MSN@TiO_2_ was chosen as this combination showed highest sorption values (Fig. 5).

##### Zwitterion

**Figure 7:**
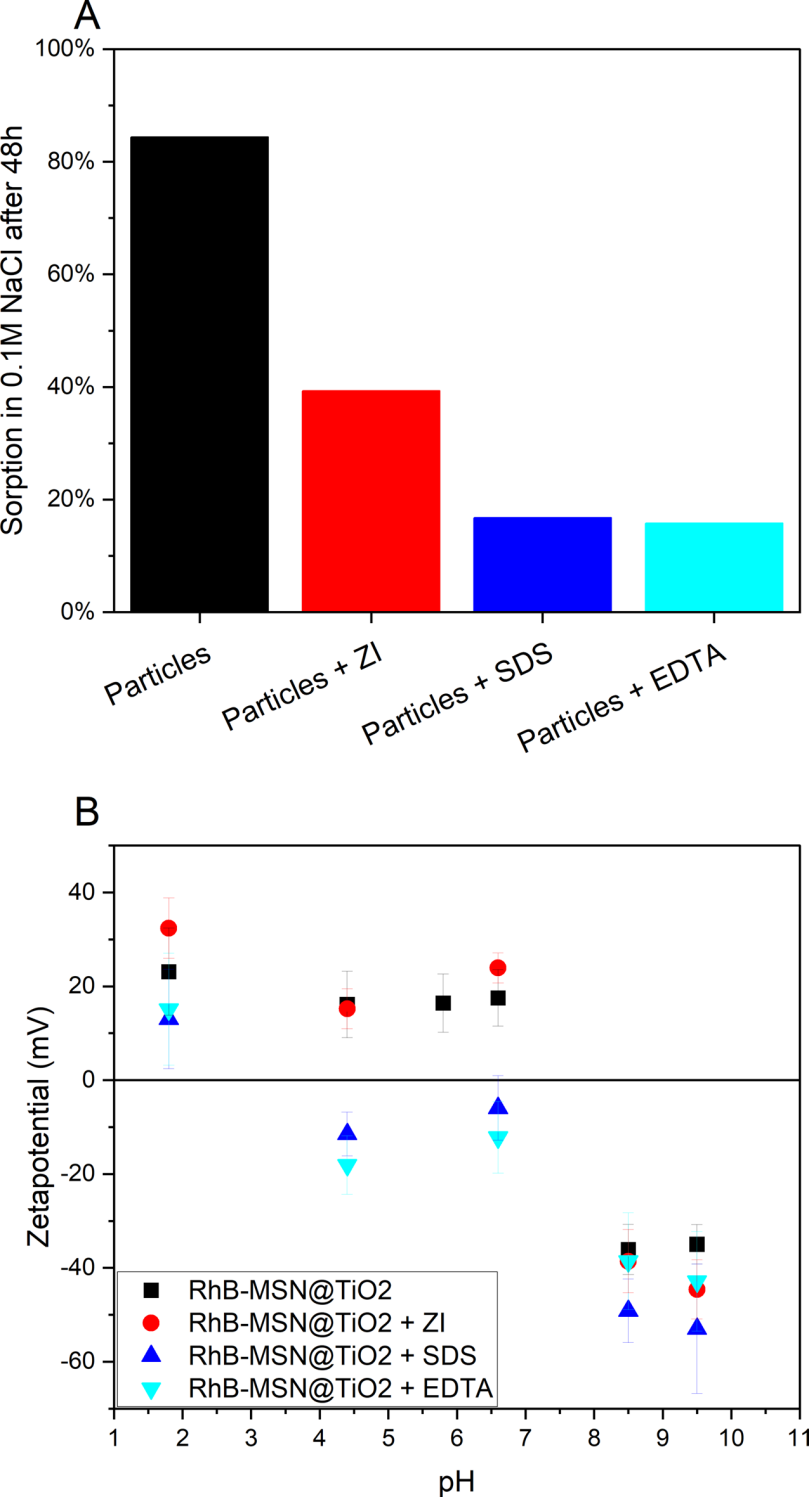
(**A**) Impact of three different additives on sorption of RhB-MSN@TiO_2_ in 0.1 M NaCl solution under batch reactor conditions. Tested additives zwitterion SB3-14 (ZI), sodium dodecyl sulfate (SDS) and ethylenediaminetetraacetic acid (EDTA) proved to be effective in lowering sorption affinity towards quartz. (**B**) Impact of additives on ζ-potential of RhB-MSN@TiO_2_. Addition of SDS and EDTA significantly reduce ζ-potential in acidic to near neutral pH ranges.

The tested zwitterionic additive was least effective in lowering sorption affinity, but still sorption was reduced to 39% and K_D_ valued of 1.62 after 48 h which is lower than sorption and K_D_ of unmodified RhB-MSN@TiO_2_ (84% and 16.28, respectively). The ζ-potential at pH 6.6 of RhB-MSN@TiO_2_ with ZI is 23.9 mV ± 3.2 mV and therefore slightly more positive than without ZI (17.5 mV ± 6 mV) (Fig. 7B). Zwitterions are not expected to affect ζ-potential because the molecule carrying positive and negative charges can exhibit both sides to particles and/or surrounding. However, attached molecules can affect interparticle forces^55^ thus possibly increasing repulsive forces, and consequently lowered sorption affinity is observed.

##### SDS

Addition of SDS lowered the sorption affinity significantly; within 48 h only 17% of the particles were sorbed from the solution compared to 84% sorption without addition of SDS (Fig. 7A). K_D_ value is reduced to 0.5, which is also lower than K_D_ of free dye (0.99). Despite improving colloidal stability^36^, the anionic surfactants altered the ζ-potential; attachment of SDS molecules shifted the iep of the particles to more acidic (between 1.8 and 4.4, Fig. 7B). Furthermore, ζ-potential is significantly lowered compared to unmodified RhB-MSN@TiO_2_, negative at pH 6.6 and resembles that of pure MSN (Fig. 3). The colloidal stability is enhanced and sorption affinity is lowered by addition of anionic surfactant. The observed lower sorption affinity can be attributed to increased repulsive forces between particle and particle as well as between the particles and the mineral surfaces.

##### EDTA

EDTA also effectively lowered sorption affinity (16% and K_D_ 0.47 after 48 h) and the ζ-potential of the particles. The impact of EDTA is similar to SDS. Iep was shifted into the acidic region similar as through addition of SDS. EDTA is known to adsorb on titania nanoparticles improving their colloidal stability^71,72^.

All additives successfully lowered the sorption affinity of the tested RhB-MSN@TiO_2_ tracer nanoparticles. Possible explanations for this enhancement can be found in DLVO theory. On one hand, the repulsive electric double layer forces are affected by the change of the ζ-potential (Fig. 7B) leading to higher colloidal stability and possibly to a reduction in the potential difference between particles and quartz surface. On the other hand, adsorption of surfactants can build up a steric barrier^73^ (XDLVO theory), drastically increasing the repulsive forces, and are able to compensate the attractive van der Waals forces at small distances (usually twice the length of the polymeric chain). Overall, the additives are effective in reducing sorption and coagulation. As preliminary flow-through experiments suggest, this surface modification strategy is also valid for flow-through setups^74^.

### Surface modification

**Figure 8:**
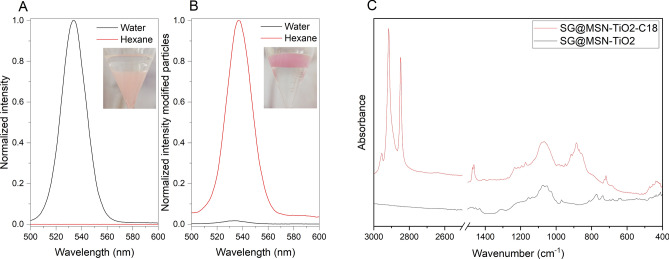
Normalized intensity of SG-MSN@TiO_2_ in water and hexane (**A**) and normalized intensity of C18 modified SG-MSN-TiO_2_ in water and hexane (**B**). Inset photographs in (**A**) and (**B**) show phase separation with bottom phase water and top phase hexane. (**C**) ATR spectra of unmodified SG-MSN@TiO_2_ and C18 modified SG-MSN@TiO_2_ particles.

Whereas additives mostly change the effective charge of the nanoparticles, the hydrophilic/hydrophobic properties of the nanoparticle surface can also be modified. In order to alter the hydrophilic character of dye-MSN@TiO_2_ particles to hydrophobic, we modified the surface by self-assembly of a hydrophobic silane monolayer (octadecyltrimethoxysilane, C18). To prove successful surface modification, non-modified particles and modified particles were dispersed in a water-hexane (oily phase) mixture. Consequently, hydrophilic (i.e. unmodified) particles were only dispersible in water, whereas hydrophobic (i.e. modified) particles were only dispersible in hexane. Figure 8A shows normalized fluorescence emission spectra of unmodified SG-MSN@TiO_2_ and modified SG-MSN@TiO_2_-C18 in water and hexane (Fig. 8). The photographs show phase-separation of water-hexane with SG-MSN@TiO_2_ (A) and SG-MSN@TiO_2_-C18 (B) (bottom phase water, top phase hexane). Fluorescence spectra confirm that while having hydrophilic particles, no fluorescence in hexane could be detected. Changing surface properties to be hydrophobic shows opposite behavior with fluorescence in hexane and minor fluorescence in water, which proofs successful surface modification. Additionally, the ATR spectra (C) of modified SG-MSN@TiO_2_-C18 reveals the presence of asymmetric and symmetric CH_2_ stretching vibrations at 2915 cm^−1^ and 2850 cm^−1^, respectively as well as CH_3_ asymmetric stretching vibration at 2950 cm^−1^ which can be assigned to the C18 monolayer.

## Conclusions

Performing tracer tests to assess and characterize reservoirs and aquifers is an established method in geosciences. However, the variety of possible applicable tracers is limited due to constraints such as degradation, sorption and/or retention of the tracer molecules. There is a need for new kind of tracer types to overcome these issues. With a multidisciplinary approach combining nanoscience, chemistry and geoscience we present an approach to develop tracer technology and present a first proof-of-concept demonstration on a laboratory scale. The introduced concept of tracer multiplicity bears great promise for geothermal and hydro-geological exploration purposes owing to the broad variety of possible applicable and distinguishable tracers. By encapsulating dye inside a nanoparticle carrier, the dye is shielded from surrounding media and can remain unaffected by harsh environments. Since the properties of tracer dye become less decisive when encapsulated, properties of nanoparticle carriers will be decisive for transport and sorption within reservoirs. Moreover, utilizing complex systems such as nanoparticles entail the advantage of its modular architecture, which offers numerous modification possibilities. An advantage, which does not exist for molecular tracers: The outer surface of the nanoparticle can be easily modified to specifically adapt the tracers to each unique challenge a certain medium may pose through its distinct combination of mineralogy, fluid composition and flow parameters. We proved that:

- Successful encapsulation and protections/shielding of different dyes inside a modular nanoparticle carrier is achievable.
- Encapsulation increases temperature resistance of organic dye molecules.
- Sorption affinity towards quartz sand can significantly be reduced for cationic dyes upon encapsulation.
- Encapsulated dyes show higher similarities in breakthrough curves compared to free dye. Despite the fact that DLVO calculations suggest higher sorption affinity for nanoparticles under static conditions, flow experiments have shown that transport is more dependent on hydrodynamic forces.
- By modifying surface properties of nanoparticles through addition of surfactants and hence altering ζ-potential the sorption affinity can lowered.

Furthermore, the presented type of nanoparticle tracer system satisfies the prerequisites for tracers of having long-term integrity, being identifiable and possessing favorable transport properties. The encapsulated dyes serve as uniquely identifiable entity while the silica nanocarrier and the titania shell act as shielding and protection against influences from the surrounding. The protective feature of the nanocapsule ensures long-term integrity and is prevented from dissolution by the stable titania layer. The favorable transport properties of nanoparticles can further be enhanced by surface modification strategies, the toolbox of nanoparticle engineering is vast and can help adapting particle properties to reservoir conditions.

This first proof-of-concept experiments on a laboratory scale have to be followed-up with implementation under increasingly more complex and realistic conditions, all the way to successful application in field experiments. Enabling simple and feasible tracer multiplicity tests could significantly extend the toolbox of tracers for geothermal and hydrological tracer tests, thereby enabling multi-well tracer tests or multi-tracer tests, thus increasing the scope of attainable information, simplifying analysis and reducing ambiguity in subsurface reservoir exploration tests.

### Supplementary Information

Supplementary Information.

## Data Availability

Data is provided within the manuscript or supplementary information files.
