# Supplementary material for: Temperature stability and enhanced transport properties by surface modifications of silica nanoparticle tracers for geo-reservoir exploration
Source: Sci Rep. 2024 Aug 19;14:19222. doi: 10.1038/s41598-024-70132-z (PMC11333493; doi:10.1038/s41598-024-70132-z)
Supplement: Supplementary file 1 — Supplementary Information. [file 41598_2024_70132_MOESM1_ESM.pdf]

Supplementary file

Temperature stability and enhanced transport properties by surface modifications of silica nanoparticle tracers for geo-reservoir exploration

Laura Spitzmüller, Jonathan Berson, Thomas Schimmel, Thomas Kohl, Fabian Nitschke

1. Synthesis

1.1. Chemicals

For synthesis the following chemicals were used: Millipore water (18MΩ), ethanol (VWR Chemicals, AnalaR Normapur, ≥99.8%), acetone (VWR Chemicals, AnalaR Normapur, ≥99.8%), %), n-hexanol (VWR GPR Rectapur), cyclohexane (VWR Chemicals, AnalaR Normapur), hexane (Carl Roth, 99%), dry acetonitrile (Merck, 99.5%), Triton X-100 (Sigma Aldrich for analysis), ammonia (NH_4OH, Merck, 28-30%), sodium hydroxide (NaOH, Merck Emsure pellets), hydrochloric acid (HCl, Honeywell Fluka 36.5-38%), sulfuric acid (H_2SO_4, Merck Supelco 98%), ethylenediaminetetraacetic acid (EDTA, Sigma Aldrich 99.4-100.6%), tetrabutyl orthotitanate (TBOT, Sigma Aldrich, synthesis grade), tetraethyl orthosilicate (TEOS, Sigma Aldrich 99%), n-octadecyltrimethoxysilane (C18, ABCR GmbH, 95%), cetrimonium bromide (CTAB, Alfa Aesar, 98%), N-tetradecyl-N,N-dimethyl-3-ammonio-1-propanesulfonate (SB3-14, "ZI", Sigma Aldrich, ≥98%), sodium dodecyl sulfate (SDS, Sigma Aldrich 99%) potassium chloride (KCl, VWR GPR Rectapur >99%) and sodium chloride (NaCl, GPR Rectapur, ≥99%). Additionally, the following dyes were used: rhodamine B (RhB, Sigma Aldrich for fluorescence), rhodamine 6G (R6G, Sigma Aldrich for fluorescence), sulforhodamine G (SG, Sigma Aldrich, dye content 60%), uranine (Sigma Aldrich for fluorescence).

1.2. Synthesis of dye-MSN@TiO_2

Synthesis of the dye-MSN@TiO_2 is described in detail in Spitzmüller, et al. [1]. In brief: To synthesize the silica nanoparticle carrier cetrimonium bromide (CTAB 109 mg) is mixed with Millipore water (54 mL) and ammonia (NH_4OH 1.194 mL) and stirred for 1 hour. Tetraethyl orthosilicate (TEOS 0.465 mL) is added dropwise and the solutions is further stirred for 5 hours. The particles undergo several washing-centrifugation cycles with water and ethanol and are dried in vacuum. CTAB template is removed via calcination at 550°C with a heating rate of 1°C/min over 6 hours. The fluorescent dye is encapsulated following a synthesis procedure of Rudolph, et al. [2] and Spitzmüller, et al. [1]. The dye is added to the particles (weight ratio 4:10) and stored overnight in a glovebox under inert nitrogen atmosphere. Then 2.5 mL dry acetonitrile per 50 mg particles is added and the particles are stirred overnight in the glovebox. The particles are retrieved by centrifugation, washed with hexane, dried in vacuum and redispersed in 4.8 mL Millipore water using a sonotrode to ensure a well dispersed solution. Titania coating was performed using a reverse water-in-oil microemulsion method as described in Spitzmüller, et al. [1]. Cyclohexane (15 mL) is mixed with n-hexanol (3.6 mL) and Triton X-100 (3.44 mL). After stirring for about 60 second, the particle dispersion and tetrabutyl orthotitanate (TBOT 307 µL) are added dropwise. After 20 minutes of stirring, H_2SO_4 (60.9 µL) is added to the formed microemulsion and stirred overnight. The particles are retrieved by addition of excess amount of acetone, followed by washing-centrifugation cycles with acetone, ethanol, and water.

1.3. Surface modification with octadecyltrimethoxysilane

The titania-coated particles were silanized with octadecyltrimethoxysilane (C18) to render their surface hydrophobic analog to a procedure described in Spitzmüller, et al. [3]. Briefly, the titania-coated particles are dried and weighted. Per 50 mg particles, dry acetonitrile (2.5 mL) is used. The particles

are redispersed in acetonitrile and n-octadecyltrimethoxysilane (0.375 mL) is added to the dispersion. After stirring for 12 hours, the coated nanoparticles are retrieved by centrifugation and washed twice with acetonitrile and hexane. Eventually, the particles are dried in vacuum.

2. Analysis

2.1. Sorption experiments - c_{peak} method

Breakthrough curves were analyzed applying the c_{peak} method to calculate v_{mean}, dispersivity and dispersion coefficient (D) according to Małoszewski and Zuber [4]:

$$\frac{D}{vx} = \frac{(1-\varphi)^2}{4 * \sqrt{\varphi \left[\ln\left(2\varphi^{3/2}\right) + 1.5 * (1-\varphi)\right] * \left[\varphi * \ln\left(2\varphi^{3/2}\right) + 1.5 * (1-\varphi)\right]}}$$

And

$$\alpha = \frac{D}{v} = \frac{D}{vx} * x$$

Mean velocity is calculated:

$$v_{mean} = \frac{x}{t_0}$$

With:

$$t_0 = t_{peak}\sqrt{1 + (3 * \frac{D}{vx})^2 + 3\frac{D}{vx}}$$

2.2. DLVO theory – Calculations

DLVO theory and colloidal stability calculations were made following [5-7].

2.2.1. Van-der-Waals force

The attractive van-der-Waals forces were calculated with:

$$V_{vdW} = -\frac{A_H r}{12h}$$

With A_H Hamaker constant, r particle radius (in m) and h separation distance (in m). The exact values for the calculations are displayed in Table S 1.

2.2.2. Electric double layer forces

The EDL forces were calculated with:

$$V_{EDL} = \pi\varepsilon_r\varepsilon_0\frac{r_1 r_2}{(r_1 + r_2)} * \left(2\zeta_1\zeta_2 \ln\left(\frac{1+e^{-\kappa h}}{1-e^{-\kappa h}}\right) + (\zeta_1^2 + \zeta_2^2) * \ln\left(1 - e^{-2\kappa h}\right)\right)$$

With ε_r relative permittivity of the medium and ε_0 absolute permittivity of vacuum, $r_{1,2}$ radii of the interacting particles (the term reduces to r_1 if interaction between particle and surfaces are calculated), $\zeta_{1,2}$ ζ-potentials of the interacting particles and h separation distance. Debye length κ^{-1} is calculated with:

$$\kappa^{-1} = \frac{1}{\sqrt{8\pi\lambda_B N_A * 10^{-24} I}}$$

With λ_B Bjerrum length (in nm), N_A Avogadro constant, I ionic strength (mol/L).

2.2.3. Colloidal stability

The colloidal stability is calculated with:

$$k_{coag} = k_0 * e^{\frac{-V_{max}}{k_B T}}$$

with V_{max} being the height of the energy barrier taken from the total DLVO interaction curve (Figure 1B), k_B Boltzmann constant, T absolute temperature and k_0 expected particle collision frequency. K_0 can be calculated with:

$$k_0 = \frac{4N_A k_B T}{3\eta}$$

With η viscosity of the medium. Finally, coagulation rate, i.e. colloidal stability is calculated with:

$$v_{coag} = k_{coag} * C^2$$

With C being the particle concentration in mol/L. Unit of v_{coag} is s^{-1}, i.e. to determine colloidal stability, v_{coag} is converted to yield the desired time (minutes, hours, months, years, etc.).

Table S 1: Values used for DLVO calculations.

Parameter	Symbol	Value	Literature
Hamaker constant	A_H	$5.35*10^{-20}$ (TiO_2-water-TiO_2)<br>$6.9*10^{-21}$ (TiO_2-water-Silica)	Bergström [8]
Particle radius	r	$75*10^{-9}$	Own data
Relative permittivity of water	ε_r	80.1	
Absolute permittivity of vacuum	ε_0	$8.85*10^{-12}$	
ζ-potential	$\zeta_{1,2}$	Variable, in V	Own data
Debye length	κ^{-1}	Depends on ionic strength	
Bjerrum length water	λ_B	0.71	
Boltzmann constant	k_B	$1.38065*10^{-23}$	
Temperature	T	293.15	

3. Supplementary data

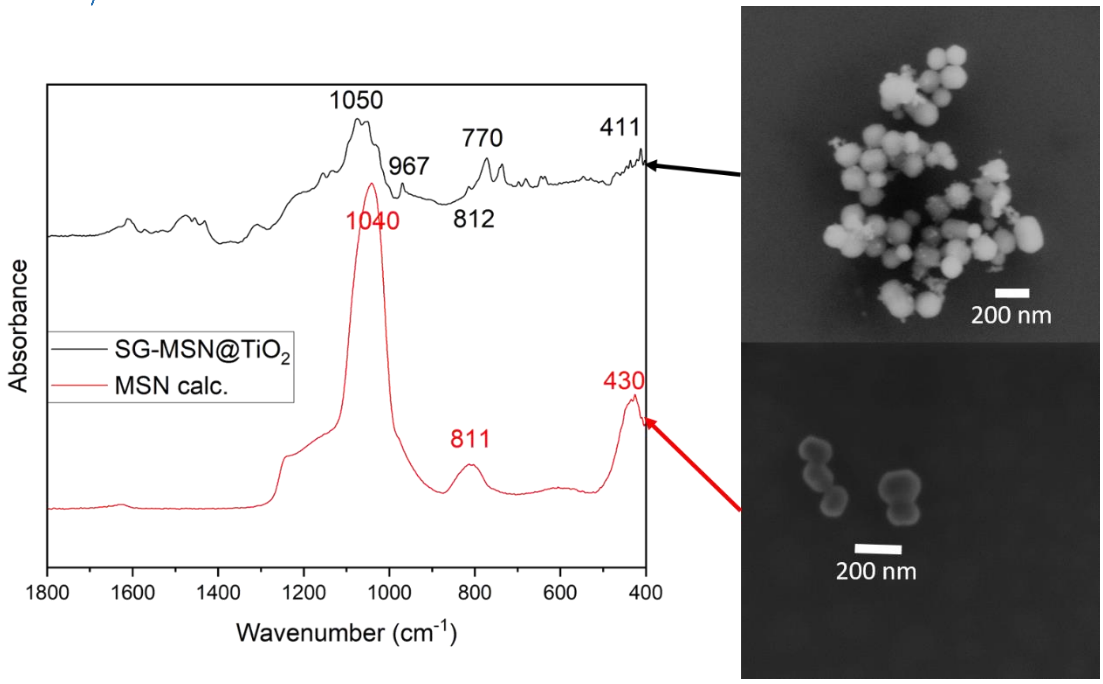

Figure S 1: Representative ATR spectra and SEM images of dye-MSN@TiO_2 and pristine MSN carrier. Wavenumber identification in Table S 2.

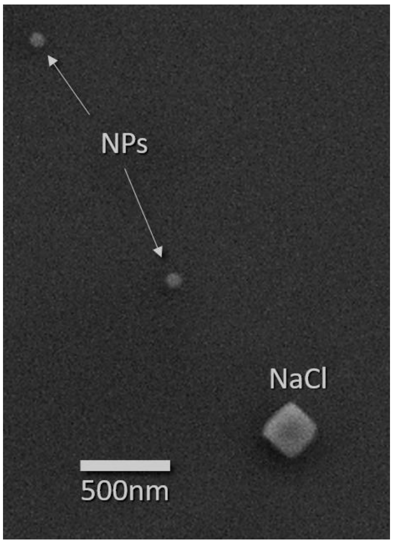

Figure S 2: SEM image of the particles after the heated experiments. The occurence of NaCl crystal is due to cooling of the 0.01M NaCl fluid used in the experiments.

Table S 2: ATR-wavenumber identifications.

Wavenumber (cm^{-1})	Identification	Literature
1050/1040	Si-O-Si	Socrates [9]
967	Ti-O-Si or Si-OH	Zu, et al. [10]
811	Si-O-Si (siloxane)	Socrates [9]
770	Ti-O str. or Ti-O-Ti bend.	Islam, et al. [11]
430	Si-O	Socrates [9]
420	Sym. Ti-O-Ti str.	Pérez, et al. [12]

Table S 3: Calculated mean velocity (m/s), dispersivitiy (m) and dispersion coefficient (cm^2/s) from the flow-through experiments displayed in Fig.4 main text.

	V_mean (10^{-4} m/s)	Dispersivity (10^{-3} m)	Dispersion coefficient (10^{-2} cm^2/s)
SG-MSN@TiO_2	3.54	6.34	2.25
SG dye	3.55	8.25	2.93
RhB-MSN@TiO_2	3.24	13.22	4.28
RhB dye	3.39	10.34	3.5
R6G-MSN@TiO_2	3.60	4.55	1.64
R6G dye	2.70	32.49	8.76

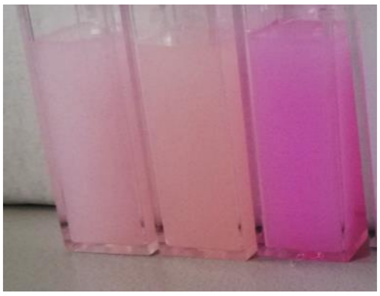

Figure S 3: Photograph of the used particle tracers in this study. From left to right: R6G-MSN@TiO_2, SG-MSN@TiO_2, RhB-MSN@TiO_2

References

1 Spitzmüller, L., Berson, J., Nitschke, F., Kohl, T. & Schimmel, T. Titania-Mediated Stabilization of Fluorescent Dye Encapsulation in Mesoporous Silica Nanoparticles. *Nanoscale Advances* **6** (2024). https://doi.org:10.1039/D4NA00242C

2 Rudolph, B., Berson, J., Held, S., Nitschke, F., Wenzel, F., Kohl, T. & Schimmel, T. Development of thermo-reporting nanoparticles for accurate sensing of geothermal reservoir conditions. *Sci Rep* **10** (2020). https://doi.org:10.1038/s41598-020-68122-y

3 Spitzmüller, L., Nitschke, F., Rudolph, B., Berson, J., Schimmel, T. & Kohl, T. Dissolution control and stability improvement of silica nanoparticles in aqueous media. *Journal of Nanoparticle Research* **25** (2023). https://doi.org:10.1007/s11051-023-05688-4

4 Małoszewski, P. & Zuber, A. On the theory of tracer experiments in fissured rocks with a porous matrix. *Journal of Hydrology* **79**, 26 (1985). https://doi.org:10.1016/0022-1694(85)90064-2

5 Agmo Hernández, V. An overview of surface forces and the DLVO theory. *ChemTexts* **9** (2023). https://doi.org:10.1007/s40828-023-00182-9

6 Worthen, A. J., Tran, V., Cornell, K. A., Truskett, T. M. & Johnston, K. P. Steric stabilization of nanoparticles with grafted low molecular weight ligands in highly concentrated brines including divalent ions. *RSC Soft Matter* **12**, 15 (2016). https://doi.org:10.1039/C5SM02787J

7 Muneer, R., Rehan Hashmet, M. & Pourafshary, P. Fine Migration Control in Sandstones: Surface Force Analysis and Application of DLVO Theory. *ACS Omega* **5**, 16 (2020). https://doi.org:10.1021/acsomega.0c03943

8 Bergström, L. Hamaker constants of inorganic materials. *Advances in Colloid and Interface Science* **70**, 45 (1997). https://doi.org:10.1016/S0001-8686(97)00003-1

9 Socrates, G. *Infrared and Raman Characteristic Group Frequencies*. Third Edition edn, (John Wiley & Sons, 2001).

10 Zu, G., Shen, J., Wang, W., Zou, L., Lian, Y. & Zhang, Z. Silica–Titania Composite Aerogel Photocatalysts by Chemical Liquid Deposition of Titania onto Nanoporous Silica Scaffolds. *ACS Appl. Mater. Interfaces* **7**, 10 (2015). https://doi.org:10.1021/am5089132

11 Islam, S., Alshoaibi, A., Bakhtiar, H., Mazher, J. & Elshikeri, N. Solvent-assisted titania nanoparticles based fiber optic pH sensor: structural, optical, and sensing characteristics. *Journal of Sol-Gel Science and Technology* **105**, 10 (2023). https://doi.org:10.1007/s10971-023-06037-6

12 Pérez, H., Miranda, R., Saavedra-Leos, Z., Zarraga, R., Alonso, P., Moctezuma, E. & Martínez, J. Green and facile sol–gel synthesis of the mesoporous SiO_2–TiO_2 catalyst by four different activation modes. *RSC Adv.* (2020). https://doi.org:10.1039/D0RA07569H
